# Serum antithrombin III as an early predictive marker for post-hepatectomy liver failure: a prospective cohort study

**DOI:** 10.1038/s41598-026-53370-1

**Published:** 2026-05-22

**Authors:** Hye-Sung Jo, Sehyeon Yu, Su-Min Jeon, Yoo-Jin Choi, Young-Dong Yu, Dong-Sik Kim

**Affiliations:** Division of HBP Surgery and Liver Transplantation, Department of Surgery, Korea University College of Medicine, Seoul, Republic of Korea

**Keywords:** Antithrombin III, Post-hepatectomy liver failure, Risk stratification, Biomarker, Biomarkers, Diseases, Gastroenterology, Medical research, Risk factors

## Abstract

Post-hepatectomy liver failure (PHLF) remains a serious complication following liver resection, yet early prediction is an unmet clinical need. This prospective study enrolled 151 patients at elevated risk of PHLF (major hepatectomy, thrombocytopenia, or hyperbilirubinemia) and evaluated serum antithrombin III (ATIII) activity, measured preoperatively and on postoperative days (PODs) 1, 2, 3, and 5, as an early predictive marker. PHLF, diagnosed according to the International Study Group of Liver Surgery criteria, occurred in 35 patients (23.2%). ATIII activity was evaluated as raw values and as the percentage change from the preoperative baseline. Raw ATIII activity was significantly lower in the PHLF group at all time points (*P* < 0.001). The POD 3 ATIII decrease was significantly greater in the PHLF group (36% vs. 29%, *P* = 0.041). Multivariable logistic regression identified ATIII change from baseline ≥ 30% at POD 3 (odds ratio 3.04, *P* = 0.021), ALBI grade B (OR 2.77, *P* = 0.031), and ICG R-15 ≥ 15% (OR 3.50, *P* = 0.034) as independent risk factors for PHLF. The multivariable model demonstrated acceptable discriminative performance (apparent AUC 0.750; bootstrap-corrected AUC 0.730). These findings suggest that early postoperative decline in ATIII activity could serve as an early biomarker for identifying patients at increased risk of PHLF, potentially enabling timely intervention.

**Supplementary Information:**

The online version contains supplementary material available at 10.1038/s41598-026-53370-1.

## Introduction

Liver resection is the primary curative treatment option for various liver tumors^1,2^. However, particularly in patients with primary liver cancer, only approximately 20% are eligible for curative surgical resection^3^. This limited applicability is largely attributable to the risk of post-hepatectomy liver failure (PHLF). This potentially fatal complication is associated with substantial morbidity and mortality, particularly in patients with compromised liver function. The reported incidence of PHLF ranges from 2 to 30%, depending on patient selection, extent of resection, and the diagnostic criteria used^4^.

The most widely adopted diagnostic criteria for PHLF, including the International Study Group of Liver Surgery (ISGLS) definition, rely on laboratory parameters measured on postoperative day 5^5^. In contrast, liver injury occurs immediately after resection because of the abrupt reduction in functional liver volume, while regenerative processes are initiated concurrently to restore hepatic function^6^. Consequently, the development of early biomarkers capable of distinguishing patients who will recover from those who will deteriorate represents a critical unmet clinical need. Although various candidate biomarkers have been proposed, none have been widely adopted in routine practice because of limited accessibility or insufficient predictive performance^7,8^.

Antithrombin III (ATIII) is a liver-synthesized glycoprotein that plays a central role in regulating the coagulation cascade^9^. Beyond its anticoagulant function, ATIII contributes to the maintenance of hepatic microcirculation and exerts anti-inflammatory effects on the vascular endothelium^10^. After liver resection, ATIII activity decreases because of reduced hepatic synthetic capacity and increased consumption, and this decline may reflect the functional recovery of the remnant liver. Previous studies have reported associations between perioperative ATIII activity and postoperative outcomes; however, the predictive value of dynamic changes in ATIII activity for PHLF has not been comprehensively evaluated^11,12^.

Accordingly, this prospective study aimed to investigate the role of serum ATIII activity as an early predictive marker for PHLF. By characterizing perioperative changes in ATIII activity and examining their association with the development of PHLF, we sought to identify a practical, early biomarker for postoperative risk stratification.

## Methods

### Study design and patient selection

This prospective study enrolled patients undergoing liver resection between September 2022 and November 2024. To identify patients at relatively high risk for clinically relevant PHLF, we included patients who met any of the following criteria: (1) major hepatectomy involving resection of three or more segments; (2) a preoperative platelet count of 100 × 10^3^/µL or less; or (3) a total bilirubin level of 1.5 mg/dL or higher in the absence of biliary obstruction—all well-established risk factors for clinically relevant PHLF^13,14^. Patients were enrolled regardless of the indication for liver resection, but were excluded if they underwent concomitant resection of other intra-abdominal organs or had coagulopathies or ongoing anticoagulation therapy that could affect ATIII activity. During the study period, none of the consecutive patients who met the inclusion criteria had any exclusion criteria, and 151 patients were ultimately enrolled.

PHLF was defined according to the ISGLS criteria as an increased INR and hyperbilirubinemia on or after postoperative day 5; in patients with preoperatively abnormal values, it was defined by a further increase in INR and serum bilirubin concentration compared with the previous day^5^. PHLF severity was graded from A to C according to its impact on clinical management. Preoperative liver function assessment routinely included evaluation for clinically significant portal hypertension based on platelet count, splenomegaly, and the presence of varices or portosystemic collateral vessels. In addition, the albumin-bilirubin (ALBI) grade and indocyanine green retention rate at 15 min (ICG R-15) were measured to provide a comprehensive evaluation of preoperative liver functional reserve. Anatomical resection was defined as the complete removal of liver parenchyma along segmental boundaries corresponding to portal venous territories. The number of resected segments was counted only when complete anatomical resection of each segment was performed. All patients provided written informed consent prior to enrollment. This study was approved by the Institutional Review Board (approval number: 2023AN0252) and conducted in accordance with the Declaration of Helsinki.

### Measurement of ATIII

Antithrombin III (ATIII) activity was measured preoperatively and on postoperative days (PODs) 1, 2, 3, and 5. For each patient, blood samples were collected at these time points to assess perioperative changes in ATIII activity. Blood was drawn gently to avoid foaming, transferred into centrifuge tubes, and centrifuged at 2000 *g* for 10 min. After centrifugation, plasma was carefully separated and stored at − 80 °C until analysis. Prior to testing, frozen samples were completely thawed at 37 °C and then equilibrated at room temperature for 15 min to ensure accurate measurement. ATIII activity was assessed using a kinetic colorimetric assay based on the antithrombin–heparin cofactor principle (Antithrombin assay kit; Roche, Germany) on an automated Cobas 8000 c702 analyzer (Roche, Germany). In this assay, heparin and excess thrombin are added to the plasma sample, allowing free antithrombin to bind thrombin and form inactive antithrombin–thrombin complexes. Residual uninhibited thrombin then cleaves the chromogenic substrate MeOCO–Gly–Pro–Arg–pNA·AcOH, releasing p-nitroaniline. The release of p-nitroaniline results in a color change detectable spectrophotometrically at 409 nm. Because the amount of residual thrombin is inversely proportional to the ATIII concentration in the sample, ATIII activity can be calculated from the rate of increase in absorbance. The analytical measurement range of this assay was 5–150%, with a reference interval of 80–120% for healthy individuals. Precision was verified by assessing repeatability and within-laboratory reproducibility under identical measurement conditions. In this study, ATIII activity was measured at all 5 planned time points in all 151 patients, with no missing data.

### Statistical analysis

Continuous variables are presented as medians with interquartile ranges (IQRs) and were compared between groups using Student’s t-test or the Mann–Whitney U test, as appropriate. Categorical variables are expressed as counts with percentages and were analyzed using the χ^2^ test or Fisher’s exact test, depending on expected frequencies. Temporal changes in ATIII activity between the PHLF and non-PHLF groups were compared using repeated-measures analysis of variance. Univariate and multivariable logistic regression analyses were performed to identify independent risk factors for PHLF. All variables with *P* ≤ 0.1 in univariate logistic regression were entered into the final multivariable model. In this study, the events-per-variable (EPV) ratio was 11.7 (35 events, 3 predictors), and multicollinearity was assessed using variance inflation factors (VIFs). Predictive performance was assessed using receiver operating characteristic (ROC) curve analysis, and the area under the curve (AUC) was calculated to evaluate discriminative ability.

Internal validation was performed using bootstrap resampling (1000 iterations) to obtain an optimism-corrected AUC. Incremental predictive value of adding POD 3 ATIII change ≥ 30% to the preoperative model was quantified using change in AUC (ΔAUC) with 95% confidence intervals by the DeLong method and continuous (rank-based) net reclassification improvement (NRI). Model calibration was assessed using the Hosmer–Lemeshow test and mean absolute calibration error (MAE). Decision curve analysis was conducted to assess the clinical utility of the prediction model by calculating net benefit across a range of threshold probabilities. A restricted cubic spline (RCS) regression model was applied to explore potential non-linear relationships between continuous variables and PHLF risk. A two-sided *P* value < 0.05 was considered statistically significant. All statistical analyses were conducted using IBM SPSS Statistics (version 24.0; IBM Corp., Armonk, NY, USA) and R software (version 4.3.3; R Foundation for Statistical Computing, Vienna, Austria) with the “rms”, “pROC”, “rmda”, “nricens” and “ggplot2” packages.

## Results

### Study population and baseline characteristics

A total of 151 patients were classified into two groups based on the ISGLS criteria: the PHLF group (n = 35, 23.2%) and the non-PHLF group (n = 116, 76.8%). Among the 35 PHLF patients, 29 (82.9%) were grade A, 4 (11.4%) grade B, and 2 (5.7%) grade C. For reference, only 3 patients (2.0%) met the 50–50 criteria for PHLF, and 1 (0.7%) met the peak bilirubin criteria. Baseline characteristics of both groups are shown in Table 1. The indication for liver resection differed significantly between the groups, with a higher proportion of patients with perihilar cholangiocarcinoma in the PHLF group than in the non-PHLF group (8 [22.9%] vs. 7 [6.0%], *P* = 0.032). The detailed diagnoses of patients classified as “Other” are presented in Supplementary Table 1. However, no significant differences were observed in underlying liver disease, including viral hepatitis, alcoholic liver disease, and cirrhosis, between groups.

**Table 1:** Baseline characteristics in the PHLF and non-PHLF groups.

	PHLF (n = 35)	non-PHLF (n = 116)	Total (n = 151)	*P* value
Baseline characteristics				
Age (years)	65 (44–76)	63 (50–71)	63 (49–73)	0.841
Sex (female)	9 (25.7%)	38 (32.8%)	47 (31.1%)	0.430
BMI (kg/m^2^)	24.2 (22.6–26.1)	24.7 (22.5–26.8)	24.6 (22.6–26.8)	0.672
PST (grade 1)^#^	3 (8.6%)	4 (3.4%)	7 (4.6%)	0.354
HTN	18 (51.4%)	53 (45.7%)	71 (47.0%)	0.551
DM	12 (34.3%)	31 (26.7%)	43 (28.5%)	0.385
CVD^#^	2 (5.7%)	7 (6.0%)	9 (6.0%)	0.944
Diagnosis				0.032
Hepatocellular carcinoma	12 (34.3%)	41 (35.3%)	53 (35.1%)	
Intrahepatic cholangiocarcinoma	5 (14.3%)	14 (12.1%)	19 (12.6%)	
Colorectal liver metastasis	5 (14.3%)	16 (13.8%)	21 (13.9%)	
Perihilar cholangiocarcinoma	8 (22.9%)	7 (6.0%)	15 (9.9%)	
Other	5 (14.3%)	38 (32.8%)	43 (28.5%)	
Underlying liver disease				
HBV	5 (14.3%)	18 (15.5%)	23 (15.2%)	0.859
HCV^#^	0 (0.0%)	3 (2.6%)	3 (2.0%)	1.000
Alcoholic liver disease^#^	0 (0.0%)	2 (1.7%)	2 (1.3%)	1.000
Cirrhosis	5 (14.3%)	18 (15.5%)	23 (15.2%)	0.859
CTP (grade B)^#^	3 (8.6%)	2 (1.7%)	5 (3.3%)	0.082
ALBI grade (≥ grade 2)	15 (42.9%)	17 (14.7%)	32 (21.2%)	< 0.001
Laboratory findings				
ICG R-15 (%)*	8.3 (5.4–16.1)	6.7 (4.2–10.6)	7.1 (4.7–11.6)	0.008
Platelets (× 10^3^/µL)	246 (199–279)	224 (176–274)	230 (182–277)	0.199
Total bilirubin (mg/dL) *	0.93 (0.72–1.40)	0.56 (0.46–0.74)	0.65 (0.47–0.88)	< 0.001
PT (INR)	1.03 (0.97–1.06)	0.98 (0.95–1.02)	0.99 (0.95–1.04)	0.063
Albumin (g/dL)	4.1 (3.7–4.5)	4.4 (4.1–4.7)	4.3 (4.0–4.6)	0.047
Creatinine (mg/dL)	0.88 (0.62–1.06)	0.81 (0.70–0.93)	0.82 (0.68–0.94)	0.515
Operation				
Number of resected segments	4 (3–5)	3 (2–4)	3 (2–4)	0.004
Minimally invasive surgery	19 (54.3%)	91 (78.4%)	110 (72.8%)	0.005
Anatomical resection	25 (71.4%)	72 (62.1%)	97 (64.2%)	0.311
Combined bile duct resection^#^	8 (22.9%)	7 (6.0%)	15 (9.9%)	0.007
Operation time (min)	290 (235–380)	265 (192–329)	270 (200–345)	0.027
Estimated blood loss (mL)	442 (162–894)	445 (177–720)	443 (167–755)	0.426
Transfusion	6 (17.1%)	6 (5.2%)	12 (7.9%)	0.032

*Mann–Whitney, ^#^Fisher’s exact.

Values are presented as median (interquartile range) for continuous data and n (%) for categorical data.

PHLF, post-hepatectomy liver failure; BMI, body mass index; PST, performance status test; HTN, hypertension; DM, diabetes mellitus; CVD, cerebrovascular disease; HBV, hepatitis B virus; HCV, hepatitis C virus; CTP, Child–Turcotte–Pugh; ALBI, albumin–bilirubin; ICG R-15, indocyanine green retention rate at 15 min; PT, prothrombin time.

Regarding preoperative laboratory findings, ICG R-15 was higher in the PHLF group than in the non-PHLF group (8.3 [IQR 5.4–16.1]% vs. 6.7 [IQR 4.2–10.6], *P* = 0.008). Furthermore, ALBI grade B or higher was more frequent in the PHLF group than in the non-PHLF group (15 [42.9%] vs. 17 [14.7%], *P* < 0.001). With respect to operative variables, the number of resected segments was higher in the PHLF group than in the non-PHLF group (4 [3–5] vs. 3 [2–4], *P* = 0.004), and minimally invasive surgery was performed less frequently in the PHLF group (19 [54.3%] vs. 91 [78.4%], *P* = 0.005). Operation time was slightly longer (290 [235–380] vs. 265 [192–329], *P* = 0.027), and intraoperative transfusion was required more often in the PHLF group than in the non-PHLF group (6 [17.1%] vs. 6 [5.2%], *P* = 0.032).

### Temporal changes in ATIII activity

**Fig. 1:**
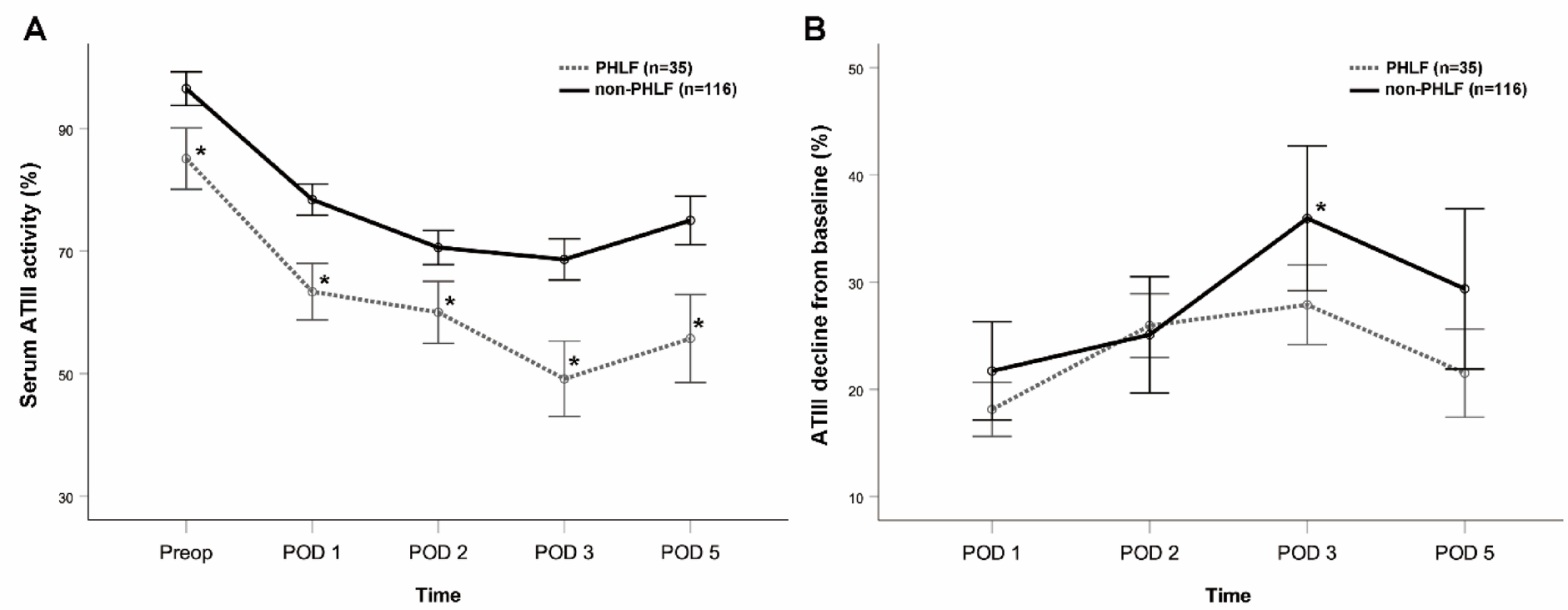
ATIII activity and changes from baseline in PHLF and non-PHLF groups. (**A**) ATIII activity showed significantly different temporal patterns between the two groups (*P* = 0.024). The PHLF group demonstrated significantly lower ATIII activity than the non-PHLF group at all time points (*P* ≤ 0.001). (**B**) Changes in ATIII activity relative to preoperative values. The magnitude of ATIII changes from baseline differed significantly between groups over time (*P* = 0.018). A significant between-group difference was observed only on POD 3 (*P* = 0.041). Asterisks indicate significant differences between groups at individual time points (*P* < 0.05). ATIII, antithrombin III; PHLF, post-hepatectomy liver failure; POD, postoperative day.

**Fig. 2:**
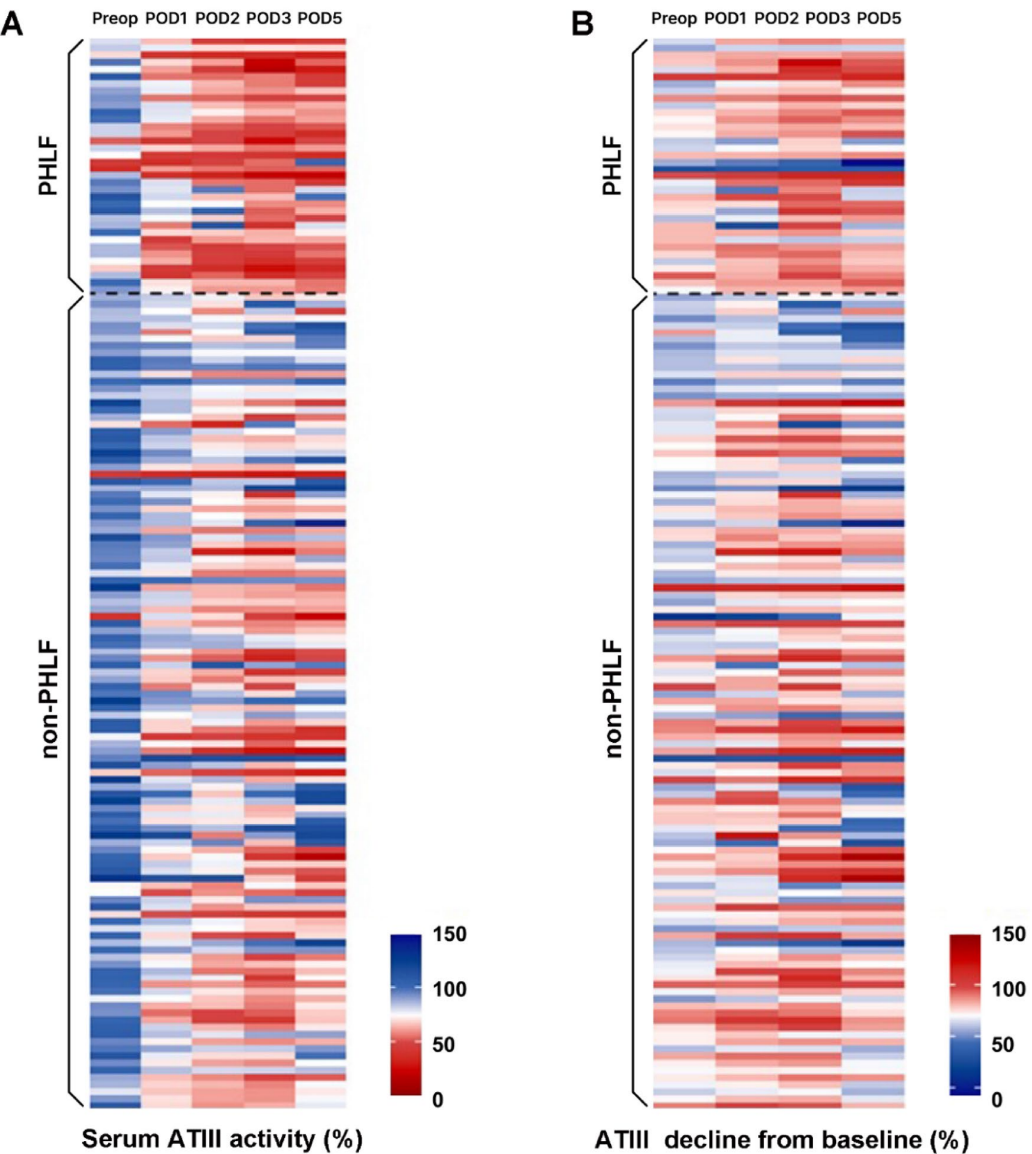
Heatmaps demonstrating individual-level temporal patterns of ATIII activity. Each row represents one patient, grouped by PHLF and non-PHLF status (separated by a dashed line). The left panel shows raw ATIII activity (%) measured preoperatively and on PODs 1, 2, 3, and 5. The right panel shows the percentage change in ATIII activity relative to the preoperative baseline at each postoperative time point. ATIII, antithrombin III; PHLF, post-hepatectomy liver failure; POD, postoperative day.

To compare serum ATIII activity between groups over time, we evaluated raw ATIII activity measured preoperatively and on PODs 1, 2, 3, and 5, as well as the percentage change from the preoperative value at each postoperative time point (Supplementary Table 2). In terms of raw values, ATIII activity at all time points, including the preoperative baseline, was significantly lower in the PHLF group than in the non-PHLF group (*P* ≤ 0.001 for all comparisons) (Fig. 1A). However, for changes from baseline, only the decrease in ATIII activity on POD 3 was significantly greater in the PHLF group than in the non-PHLF group (36% [IQR 30–45] vs. 29% [IQR 15–41], *P* = 0.041) (Fig. 1B). Figure 2 illustrates individual-level temporal patterns of ATIII activity and percentage changes from baseline using heatmaps. In Fig. 2A, the PHLF group exhibited consistently lower raw ATIII activity with a delayed postoperative recovery pattern compared with the non-PHLF group. In Fig. 2B, the PHLF group showed a greater decrease in ATIII activity, particularly on POD 3.

A more detailed assessment showed that the proportion of patients with a continued decrease in ATIII activity relative to baseline was 145 (96.0%) through POD 1, 123 (81.5%) through POD 2, 78 (51.7%) through POD 3, and 20 (13.2%) through POD 5. Among the 78 patients with a continued decrease through POD 3, PHLF developed more frequently in those with a reduction of ≥ 30% from baseline than in those with a reduction of < 30% (18/56 [32.1%] vs. 3/22 [13.6%], *P* = 0.047). To evaluate the incremental value of ATIII change at POD 3 beyond POD 1, patients were further stratified by POD 1 ATIII activity using the 60% threshold, a stratification factor adopted in the HiSCO-05 trial^12^. Among the 128 patients with POD 1 ATIII ≥ 60%, those with an ATIII change ≥ 30% at POD 3 developed PHLF more frequently than those with < 30% change (18/72 [25.0%] vs. 4/56 [7.1%], odds ratio 4.33, 95% CI 1.37–13.66, *P* = 0.009). Notably, of the 22 PHLF cases in this low-risk group, 18 (81.8%) were reclassified as high-risk by the POD 3 criterion.

When stratified by ISGLS grade, the prevalence of ATIII change ≥ 30% at POD 3 increased across the severity spectrum: 58 of 116 (50.0%) in the non-PHLF group, 22 of 29 (75.9%) in grade A, and 6 of 6 (100%) in grade B or above (*P* = 0.001). Among 64 patients with ATIII change < 30% at POD 3, none developed PHLF of grade B or above (negative predictive value 100%). Given that only 6 clinically significant events occurred, these findings are descriptive.

### Early postoperative outcomes stratified by POD 3 ATIII change

Patients with ≥ 30% ATIII decrease at POD 3 (n = 86) had a significantly higher PHLF rate than those with < 30% decrease (n = 65) (28 [32.6%] vs. 7 [10.8%], *P* = 0.002) (Table 2). Postoperative intensive care unit admission showed a trend toward higher frequency in the ≥ 30% decrease group (12 [14.0%] vs. 3 [4.6%], *P* = 0.057), and median hospital stay was significantly longer (12 [8–19] vs. 9 [8–13] days, *P* = 0.004). Major complication (Clavien–Dindo grade ≥ IIIA) rates were comparable between the groups (17 [19.8%] vs. 8 [12.3%], *P* = 0.222). Both 30-day and 90-day mortality rates were similar between groups (2 [2.3%] vs. 2 [3.1%], *P* = 1.000, and 4 [4.7%] vs. 2 [3.1%], *P* = 0.700, respectively). However, both deaths in the < 30% decrease group were attributable to pneumonia with sepsis in patients without PHLF, indicating that these events were unrelated to hepatic functional deterioration.

**Table 2:** Early postoperative outcomes stratified by POD 3 ATIII change.

	≥ 30% decrease (n = 86)	< 30% decrease (n = 65)	Total (n = 151)	*P* value
PHLF	28 (32.6%)	7 (10.8%)	35 (23.2%)	0.002
Major complication (Clavien–Dindo grade ≥ IIIA)	17 (19.8%)	8 (12.3%)	25 (16.6%)	0.222
Biliary complication^#^	6 (7.0%)	6 (9.2%)	12 (7.9%)	0.612
Bleeding complication^#^	4 (4.7%)	0 (0%)	4 (2.6%)	0.135
Infectious complication^#^	2 (2.3%)	4 (6.2%)	6 (4.0%)	0.403
ICU admission	12 (14.0%)	3 (4.6%)	15 (9.9%)	0.057
Hospital stay (days)*	12 (8–19)	9 (8–13)	11 (8–15)	0.004
30-day mortality^#^	2 (2.3%)	2 (3.1%)	4 (2.6%)	1.000
90-day mortality^#^	4 (4.7%)	2 (3.1%)	6 (4.0%)	0.700

Values are presented as median (interquartile range) for continuous data and n (%) for categorical data.

*Mann–Whitney, ^#^Fisher’s exact.

PHLF, post-hepatectomy liver failure; ICU, intensive care unit; ATIII, antithrombin III; POD, postoperative day.

### Risk factors for PHLF

**Fig. 3:**
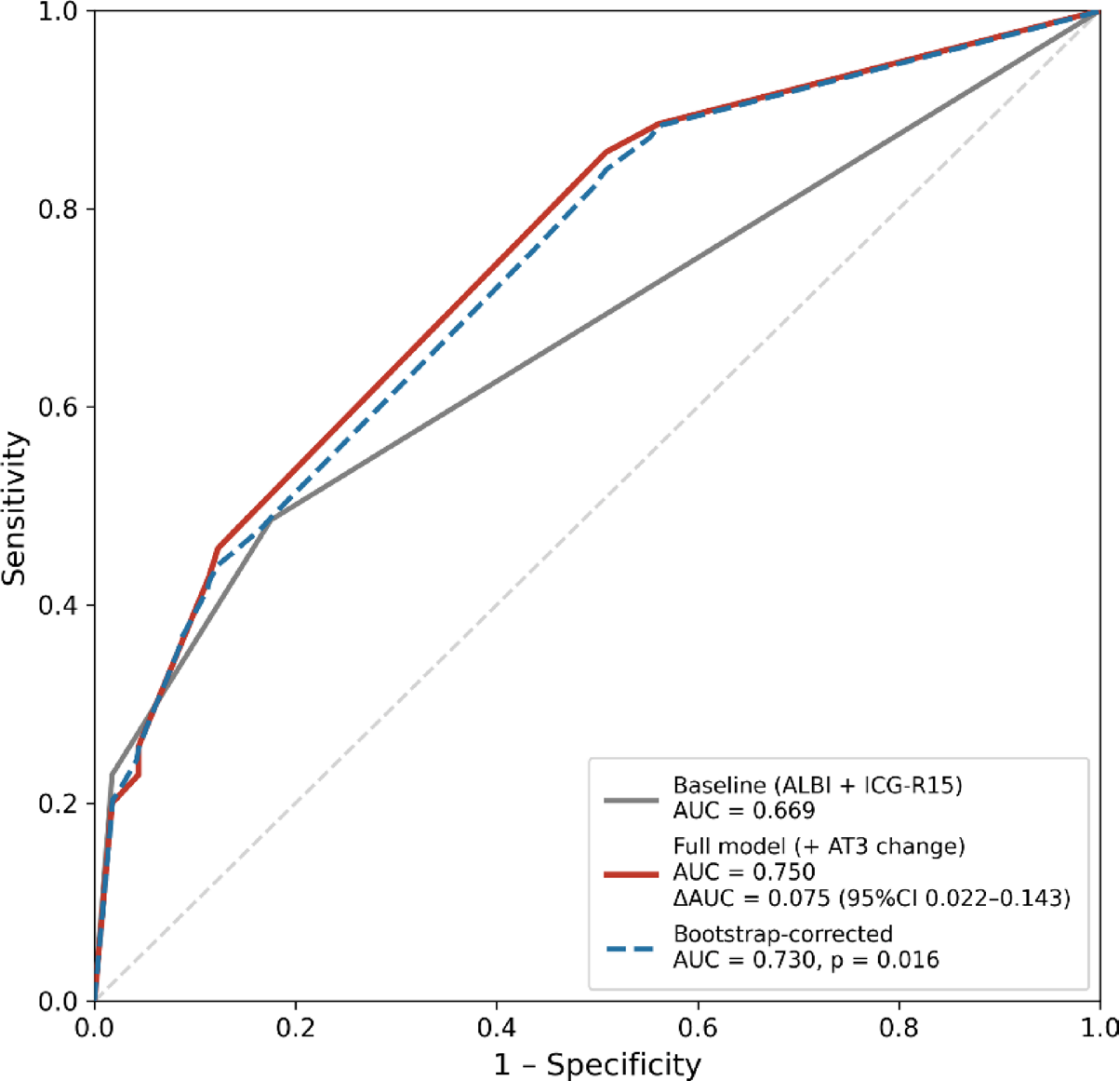
ROC curves with bootstrap internal validation based on the multivariable logistic regression model for predicting PHLF. The grey solid line represents the baseline preoperative model comprising ALBI grade and ICG-R15 alone (AUC = 0.669). The red solid line represents the full model incorporating ATIII change ≥ 30% at POD 3, in addition to the preoperative markers (AUC = 0.750; ΔAUC =  + 0.075, 95% CI 0.022–0.143, DeLong *P* = 0.016). The blue dashed line represents the bootstrap-corrected ROC curve (optimism-corrected AUC = 0.730), estimated using 1000 bootstrap resamples. The grey diagonal dashed line represents the no-discrimination reference line (AUC = 0.5). ROC, receiver operating characteristic curve; AUC, area under the curve; PHLF, post-hepatectomy liver failure.

Multivariable logistic regression analysis was performed to evaluate risk factors for PHLF (Table 3). ATIII changes from baseline ≥ 30% at POD 3 (odds ratio 3.04; 95% confidence interval 1.18–7.80; *P* = 0.021), ALBI grade B (2.77 [1.10–7.02], *P* = 0.031), and ICG R-15 ≥ 15% (3.50 [1.10–11.17], *P* = 0.034) were identified as independent risk factors for PHLF. VIFs were < 1.5 for all predictors (ALBI grade: 1.39, ICG-R15: 1.42, ATIII change at POD 3: 1.08), confirming the absence of multicollinearity. Spearman correlation analysis showed minimal correlations between ATIII change at POD 3 and ALBI or ICG-R15 (Supplementary Table 3). The apparent AUC of the multivariable model was 0.750, and bootstrap resampling (1000 iterations) yielded an optimism-corrected AUC of 0.730 (Fig. 3). We further compared the discriminative ability of individual preoperative markers, composite scores, and multivariable models (Supplementary Fig. 1). Preoperative ATIII (AUC = 0.709) performed comparably to the Model for End-Stage Liver Disease (MELD) score (0.711) and numerically outperformed ALBI (0.685), ICG-R15 (0.648), the ALBI–ICG-R15 evaluation (ALICE) score (0.688), the aspartate aminotransferase-to-platelet ratio index (APRI) (0.513), and APRI + ALBI (0.637).

**Table 3:** Univariate and multivariable logistic regression analyses for PHLF.

	Univariate analysis		Multivariable analysis	
	OR (95% CI)	*P* value	OR (95% CI)	*P* value
Age (≥ 65 years)	1.30 (0.61–2.78)	0.493		
Sex (male)	1.41 (0.60–3.30)	0.431		
BMI (< 18.5 kg/m^2^)	0.82 (0.09–7.62)	0.864		
PST (grade 1)	2.63 (0.56–12.34)	0.222		
Diagnosis (HCC)	0.95 (0.43–2.11)	0.908		
Cirrhosis	0.91 (0.31–2.65)	0.859		
ALBI grade (B)	4.37 (1.88–10.16)	0.001	2.77 (1.10–7.02)	0.031
ICG R-15 (≥ 15%)	6.23 (2.16–17.96)	0.001	3.50 (1.10–11.17)	0.034
Platelets (< 100 × 10^3^/µL)	0.65 (0.02–27.10)	0.818		
Number of resected segments	1.60 (1.15–2.23)	0.006	1.06 (0.67–1.68)	0.804
Minimally invasive surgery	0.33 (0.15–0.73)	0.006	0.62 (0.24–1.60)	0.327
Anatomical resection	1.53 (0.67–3.48)	0.313		
Combined bile duct resection	4.61 (1.54–13.84)	0.006	1.47 (0.33–6.57)	0.611
Operation time (≥ 240 min)	1.73 (0.61–4.92)	0.301		
Transfusion	3.79 (1.14–12.64)	0.030	1.54 (0.36–6.62)	0.565
ATIII activity change from baseline				
POD 1 (≥ 30%)	0.87 (0.27–2.81)	0.814		
POD 2 (≥ 30%)	2.09 (0.97–4.50)	0.059	1.43 (0.51–4.00)	0.586
POD 3 (≥ 30%)	4.00 (1.62–9.89)	0.003	3.04 (1.18–7.80)	0.021
POD 5 (≥ 30%)	3.10 (1.41–6.78)	0.005	1.31 (0.47–3.60)	0.605

PHLF, post-hepatectomy liver failure; OR, odds ratio; CI, confidence interval; BMI, body mass index; PST, performance status test; HCC, hepatocellular carcinoma; ALBI, albumin–bilirubin; ICG R-15, indocyanine green retention rate at 15 min; ATIII, antithrombin III; POD, postoperative day.

**Fig. 4:**
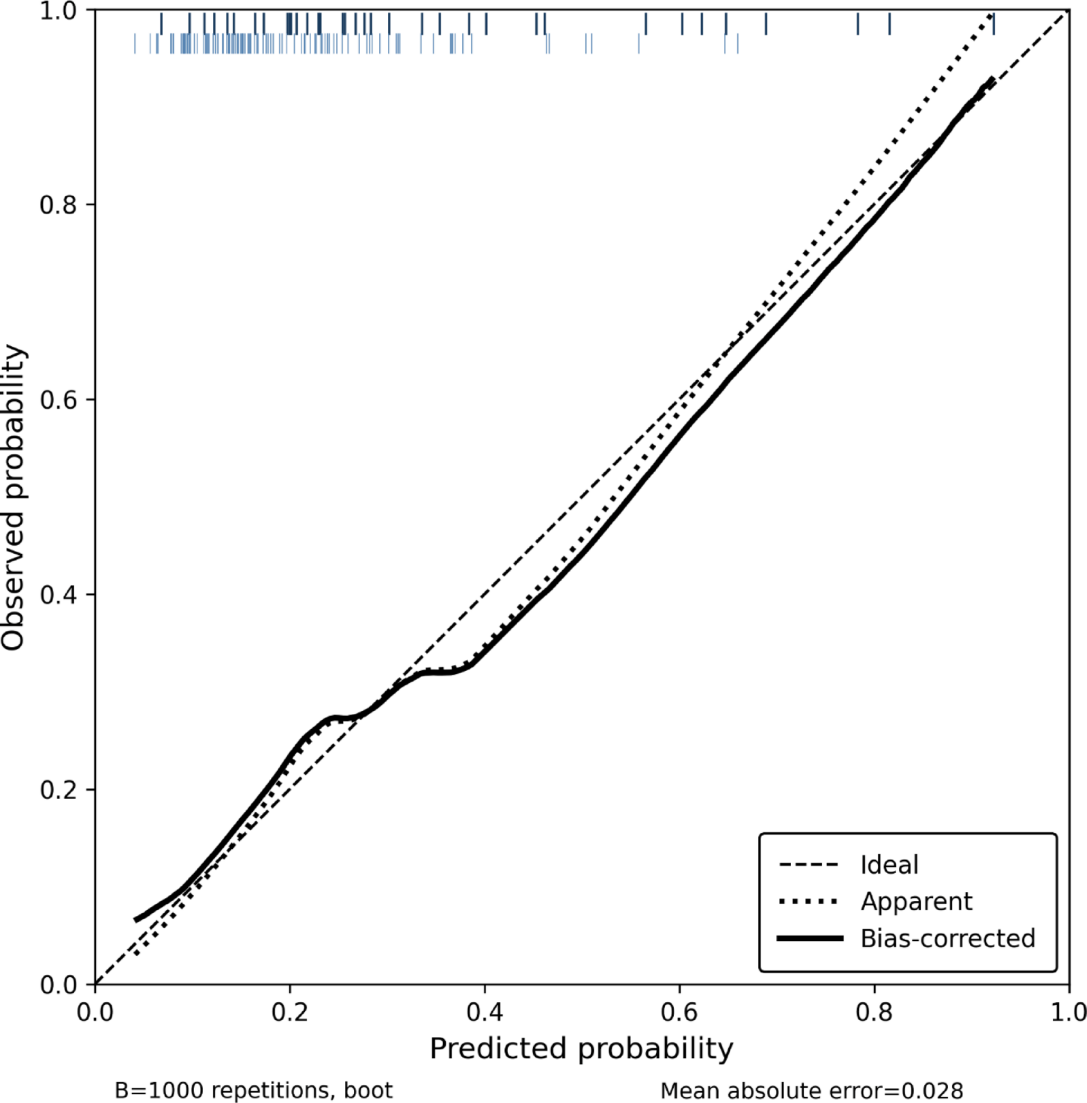
Calibration plot of the multivariable prediction model for PHLF. The dashed diagonal line represents ideal calibration (perfect agreement between predicted and observed probabilities). The dotted curve represents the apparent calibration curve, and the solid curve represents the bias-corrected calibration curve estimated using 1000 bootstrap resamples. The rug plot along the upper margin indicates the distribution of predicted probabilities among non-PHLF patients. The Hosmer–Lemeshow goodness-of-fit test demonstrated satisfactory calibration (χ^2^ = 6.12, *P* = 0.737), with a mean absolute calibration error of 0.028. PHLF, post-hepatectomy liver failure.

**Fig. 5:**
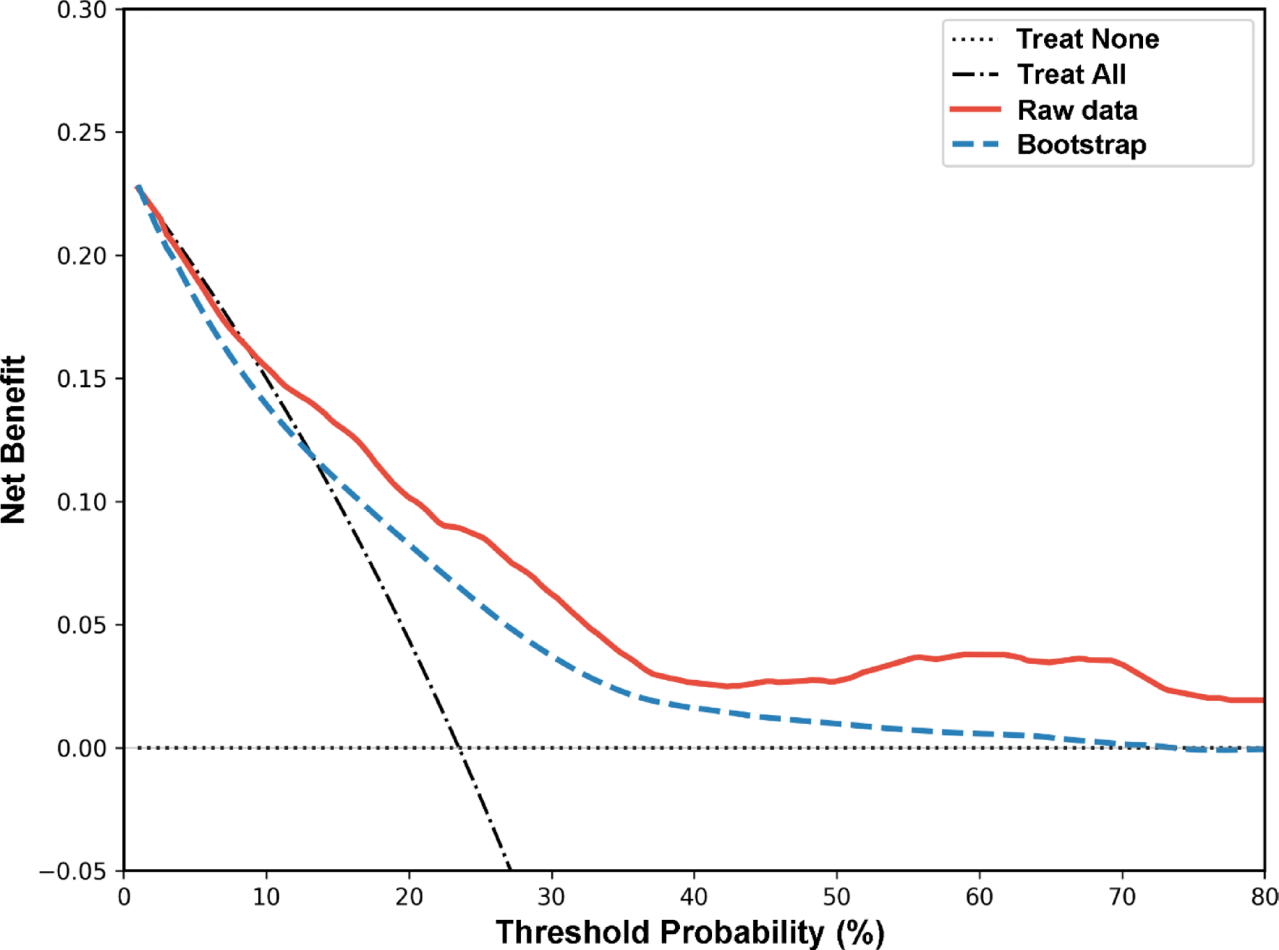
Decision curve analysis for multivariable prediction model. Decision curve analysis evaluating the clinical utility of the multivariable prediction model across a range of threshold probabilities. The solid red line represents the apparent model, and the dashed blue line represents the bootstrap-validated model. The dash-dotted line indicates the “treat all” strategy, and the dotted line indicates the “treat none” strategy. Both models demonstrated positive net benefit compared with default strategies across threshold probabilities of approximately 10–50%, supporting the clinical applicability of the model for identifying high-risk patients.

The incremental value of adding POD 3 ATIII change ≥ 30% to the preoperative model (ALBI + ICG-R15) was statistically significant: ΔAUC =  + 0.075 (95% CI 0.022–0.143, *P* = 0.016; Fig. 3) and a continuous NRI of + 0.583 (95% CI 0.257–0.895, *P* < 0.001) (Supplementary Table 4). Model calibration was satisfactory: Hosmer–Lemeshow χ^2^ = 6.12 (*P* = 0.737), MAE = 0.028 (Fig. 4). Decision curve analysis showed a positive net benefit relative to “treat-all” and “treat-none” strategies across threshold probabilities of 10–50%, supporting the potential clinical utility of the model (Fig. 5). In a sensitivity analysis excluding patients with cirrhosis (n = 128), ATIII change ≥ 30% at POD 3 remained a significant predictor of PHLF (odds ratio 3.29 [1.30–8.32], AUC 0.739).

**Fig. 6:**
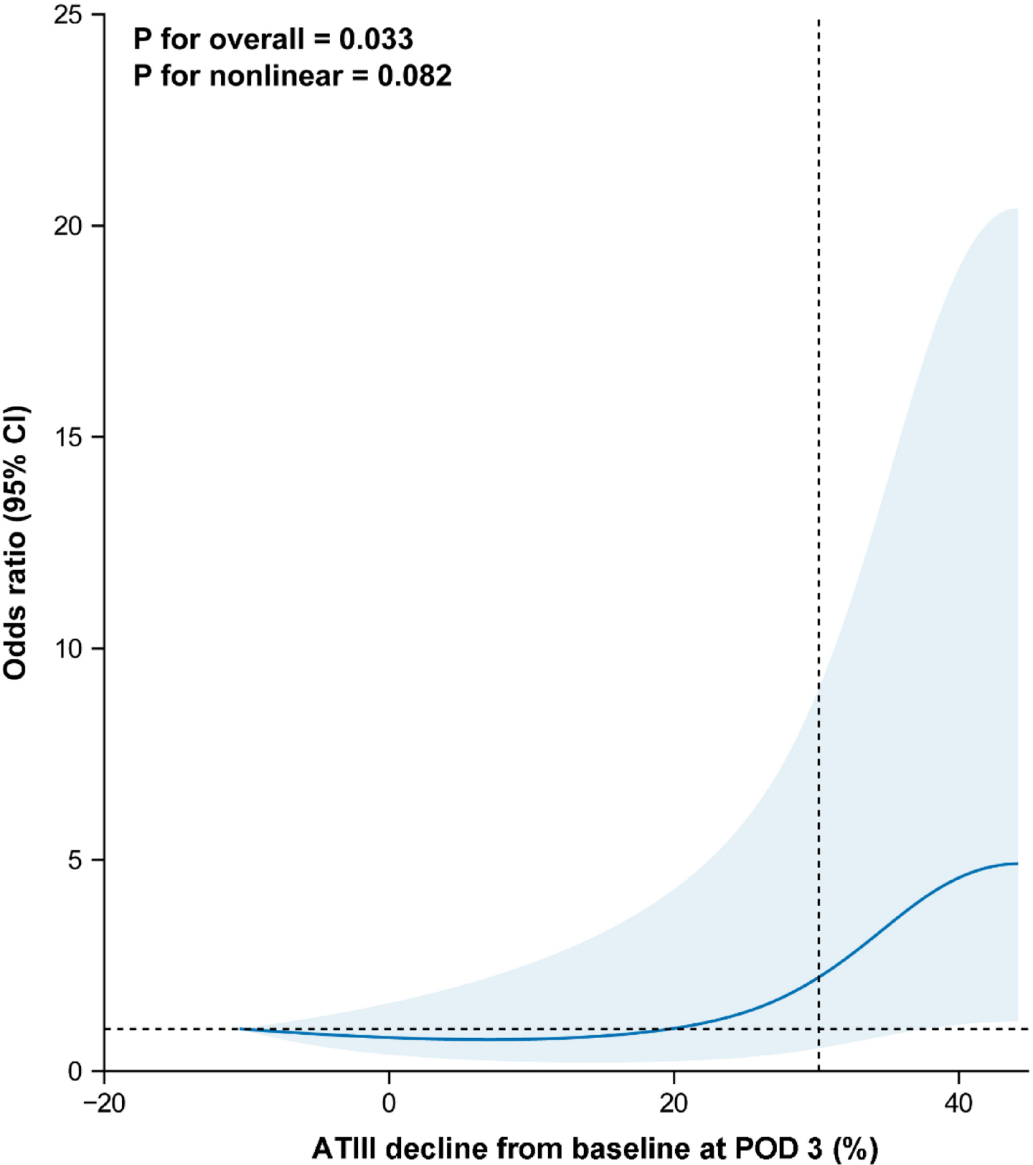
Restricted cubic spline curve showing the association between ATIII change from baseline on POD 3 and the risk of PHLF, adjusted for other risk factors identified in multivariable logistic regression analysis (ALBI grade and ICG R-15). The solid line represents the odds ratio, and the shaded area indicates the 95% confidence interval. The dashed horizontal line indicates an odds ratio of 1 (reference). The risk of PHLF increased as ATIII activity on POD 3 declined more relative to baseline (*P* for overall = 0.033; *P* for nonlinearity = 0.082). ATIII, antithrombin III; POD, postoperative day; PHLF, post-hepatectomy liver failure; ALBI, albumin-bilirubin; ICG R-15, indocyanine green retention rate at 15 min.

A scatter plot was used to illustrate the relationship between absolute ATIII activity and the percentage decline from baseline (Supplementary Fig. 2). POD 3 ATIII activity was strongly and inversely correlated with the percentage decline from baseline (r = − 0. 79, *P* < 0.001). When a high-risk zone was defined as POD 3 ATIII < 50% with a decline ≥ 30%, 42.9% (15/35) of patients in the PHLF group fell within this zone compared with 14.7% (17/116) in the non-PHLF group. Figure 6 shows an RCS curve illustrating the association between ATIII change from baseline at POD 3 and the odds ratio for PHLF, adjusted for the other risk factors identified in the multivariable analysis (ALBI grade and ICG R-15). The risk of PHLF increased progressively as ATIII activity on POD 3 declined more relative to baseline.

## Discussion

PHLF remains one of the most critical complications following liver resection. Unlike other organs, the feasibility of liver resection is determined not only by the extent of resection based on morphologic tumor burden but also by underlying liver function and the expected functional reserve of the remnant liver. The most widely adopted diagnostic criteria for PHLF are the ISGLS definition^5^. The ISGLS definition evaluates PHLF based on serum bilirubin and prothrombin time levels on POD 5. Its distinguishing characteristic is its grading system, which assesses severity according to the degree of deviation from routine postoperative management and the need for invasive treatment. The increasing adoption of minimally invasive surgery and parenchyma-sparing limited resection strategies has likely contributed to a gradual decline in the incidence of severe PHLF^15^. Nevertheless, accumulating evidence indicates that PHLF affects both short-term recovery and long-term outcomes^16,17^; therefore, early and effective prediction of PHLF is a critical first step toward overcoming this serious complication.

A common feature of established PHLF diagnostic criteria is that a definitive diagnosis cannot be made until at least POD 5^5,18,19^. This is because bilirubin and prothrombin time levels in the early postoperative period do not reliably distinguish patients whose liver function will recover from those who will deteriorate. This limitation has been consistently observed in both derivation cohorts and subsequent validation studies^17,20^, underscoring the difficulty of advancing the timing of PHLF diagnosis earlier than POD 5. Nevertheless, liver regeneration begins immediately after surgery, as hepatocyte proliferation and restoration of hepatic microcirculation are initiated early in response to the abrupt reduction in functional liver volume^21^. In our study, we demonstrated that early postoperative change in ATIII activity could predict PHLF. Multivariable regression analysis identified a ≥ 30% reduction in ATIII activity from baseline at POD 3 as an independent risk factor for PHLF.

Furthermore, restricted cubic spline analysis demonstrated that the odds of PHLF increased progressively with greater reductions in ATIII activity at POD 3, supporting a dose–response relationship between early ATIII decline and subsequent PHLF development. The discriminative performance of the multivariable model remained acceptable after bootstrap internal validation. In addition, decision curve analysis demonstrated a positive net benefit across clinically relevant threshold probabilities, supporting the potential clinical utility of ATIII-based risk stratification for identifying high-risk patients who may benefit from early intervention. As a predictive marker for PHLF, ATIII offers several practical advantages: it can be readily measured using standard laboratory platforms and has potential therapeutic implications. Moreover, unlike static preoperative markers, serial ATIII measurements reflect the dynamic trajectory of postoperative liver function, enabling individualized risk assessment. In the present cohort, preoperative ATIII demonstrated discriminative performance comparable to MELD and marginally superior to ALBI, ICG-R15, and composite scores including ALICE and APRI + ALBI. The relatively lower performance of APRI + ALBI may reflect differences in cohort composition compared with the predominantly colorectal liver metastasis cohorts in which this score was developed^22,23^. Importantly, the addition of the POD 3 ATIII change to the preoperative model significantly improved predictive performance, supporting a dual role of ATIII as both a preoperative screening tool and a perioperative real-time marker.

ATIII is a liver-synthesized glycoprotein that serves as the primary endogenous inhibitor of thrombin and other activated serine proteases in the coagulation cascade, accounting for approximately 80% of thrombin inactivation in blood^24^. Following liver resection, ATIII activity declines because of reduced hepatic synthetic capacity, consumption in the setting of surgical stress and systemic inflammation, and altered transcapillary distribution. This decrease creates a procoagulant state that promotes intrahepatic microvascular thrombosis, leading to sinusoidal obstruction and impaired hepatic microcirculation. Liver sinusoidal endothelial cells are particularly vulnerable to ischemia–reperfusion injury following hepatectomy, and subsequent endothelial damage facilitates platelet aggregation and fibrin deposition within the sinusoids^25^. Such microcirculatory disturbances compromise oxygen and nutrient delivery to the regenerating liver in the early postoperative period, thereby impairing hepatic functional recovery. Beyond its canonical anticoagulant function, ATIII also exerts direct anti-inflammatory and endothelial-protective effects through interactions with the vascular glycocalyx^26^, underscoring its potential role in maintaining hepatic microvascular integrity after liver resection.

Several previous studies have investigated the relationship between ATIII and postoperative outcomes following liver resection^11,12,27–29^. Preoperative ATIII levels have been identified as a significant prognostic factor for overall and disease-free survival in patients with hepatocellular carcinoma undergoing curative hepatectomy^29^. However, these studies relied on a single preoperative ATIII value and did not account for variations in surgical extent or postoperative recovery. Another study demonstrated that ATIII activity on POD 1 was a valuable predictor of postoperative liver dysfunction in patients with colorectal liver metastases^27^. While prior studies primarily focused on preoperative or early postoperative ATIII activity, our study extends this evidence by evaluating the predictive value of perioperative changes in ATIII activity for PHLF. This approach enabled assessment of serial changes in ATIII activity during postoperative recovery and identified the magnitude of ATIII decrease on POD 3 as a key predictor of PHLF.

To date, no pharmacologic agents have been established to directly improve liver function beyond supportive management for PHLF^4^. Consequently, during the immediate postoperative period—when the remnant liver is struggling for volumetric and functional regeneration—realistic management focuses on early identification and correction of potentially reversible insults, including infectious complications, cardiopulmonary compromise, and renal dysfunction. This supportive approach aims to preserve hepatic perfusion, minimize systemic and hepatic metabolic stress, and maintain conditions that allow sufficient time for liver regeneration^30^. Given the role of ATIII in hepatic microcirculation and coagulation, it could be considered a therapeutic target. Experimental studies have demonstrated that ATIII supplementation improves hepatic energy status and microcirculation after ischemia–reperfusion injury, supporting a mechanistic connection between ATIII deficiency and impaired postoperative recovery^31,32^. Clinical data also indicate that postoperative ATIII administration could attenuate PHLF in patients with hepatocellular carcinoma by mitigating postoperative coagulopathy^11^. Nevertheless, a multicenter randomized trial in Japan failed to demonstrate a significant preventive effect of ATIII supplementation on PHLF incidence^12^. That negative result should be interpreted cautiously, as ATIII was administered without risk stratification based on ATIII activity, and concomitant use of gabexate mesylate may have confounded the results. In our study, early detection of a significant postoperative decline in ATIII activity could identify high-risk patients before PHLF becomes clinically apparent, providing a window for timely intensive monitoring and therapeutic intervention. Based on these findings, prospective trials are warranted to externally validate ATIII as a predictive marker for PHLF and to evaluate whether targeted ATIII supplementation in a high-risk population identified by serial ATIII monitoring can improve postoperative outcomes.

This study has several limitations. First, it was a single-center study with a relatively small sample size; however, the prospective design and standardized perioperative sampling protocol strengthen reliability. Second, the cohort included patients undergoing hepatectomy for various indications with different functional and volumetric hepatic reserve. Although this heterogeneity may introduce clinical variability, it also reflects real-world practice and supports the broader applicability of ATIII as an early marker for PHLF. Third, PHLF was defined using ISGLS criteria; grade A events, which accounted for 82.9% of PHLF cases, have a limited clinical impact. At the study design stage, we anticipated that clinically significant PHLF (grade B or above) would be rare in contemporary practice and therefore enrolled a cohort at elevated risk for PHLF. Despite these efforts, PHLF grade B or above was observed in only 6 patients (4.0%), precluding dedicated multivariable modeling. Nevertheless, the increasing prevalence of ATIII change ≥ 30% at POD 3 across the severity spectrum suggests a potential association across PHLF grades, although its clinical relevance should be interpreted with caution. A multicenter prospective cohort study is currently being planned to enroll sufficient grade B or above events. Finally, as this cohort was restricted to patients at elevated risk of PHLF, the role of routine ATIII surveillance in low-risk patients remains to be defined.

## Conclusions

The decline in serum ATIII activity on POD 3 was an independent predictor of PHLF in this prospective study, potentially enabling earlier identification of patients at increased risk. Larger multicenter studies are warranted to validate these findings and to evaluate ATIII supplementation as a potential therapeutic strategy for PHLF.

## Supplementary Information

Below is the link to the electronic supplementary material.

Supplementary Material 1

## Data Availability

The datasets used and analyzed during the current study are available from the corresponding author on reasonable request.
