# Supplementary material for: Serum antithrombin III as an early predictive marker for post-hepatectomy liver failure: a prospective cohort study
Source: Sci Rep. 2026 May 22;16:23307. doi: 10.1038/s41598-026-53370-1 (PMC13402788; doi:10.1038/s41598-026-53370-1)
Supplement: Supplementary file 1 — Supplementary Material 1 [file 41598_2026_53370_MOESM1_ESM.docx]

**Supplementary Table 1** Detailed diagnoses of patients classified as "Other" in Table 1

| Diagnosis | PHLF (n=5) | non-PHLF (n=38) | Total (n=43) |
| --- | --- | --- | --- |
| Living liver donor | 3 | 25 | 28 |
| Intrahepatic duct stone | 1 | 4 | 5 |
| Chronic cholangitis | 0 | 3 | 3 |
| IPNB | 0 | 2 | 2 |
| Gallbladder carcinoma | 0 | 1 | 1 |
| Neuroendocrine tumor | 0 | 1 | 1 |
| Angiomyolipoma | 0 | 1 | 1 |
| Focal nodular hyperplasia | 1 | 0 | 1 |
| Mucinous cystic neoplasm | 0 | 1 | 1 |

PHLF, post-hepatectomy liver failure; IPNB, Intraductal papillary neoplasm of the bile duct.

**Supplementary Table 2** Raw ATIII activity and percentage changes from baseline in the PHLF and non-PHLF groups

|  | PHLF (n=35) | non-PHLF (n=116) | Total (n=151) | *P* value |
| --- | --- | --- | --- | --- |
| **Raw ATIII activity** |  |  |  |  |
| Baseline (%) | 86 (76–98) | 96 (88–106) | 94 (86–104) | 0.001 |
| POD 1 (%) | 64 (52–78) | 80 (69–86) | 78 (66–84) | <0.001 |
| POD 2 (%) | 56 (47–66) | 71 (61–79) | 67 (58–77) | <0.001 |
| POD 3 (%) | 53 (40–59) | 66 (57–83) | 62 (53–75) | <0.001 |
| POD 5 (%) | 54 (46–74) | 72 (62–90) | 70 (56–84) | <0.001 |
| **ATIII activity change from baseline** |  |  |  |  |
| POD 1 (%) | 24 (12–30) | 17 (9–26) | 18 (10–28) | 0.177 |
| POD 2 (%) | 31 (17–36) | 26 (16–33) | 27 (17–33) | 0.785 |
| POD 3 (%) | 36 (30–45) | 29 (15–41) | 32 (18–42) | 0.041 |
| POD 5 (%) * | 34 (21–44) | 23 (8–35) | 26 (11–37) | 0.071 |

^*^ Mann–Whitney

Values are presented as median (interquartile range) for continuous data and n (%) for categorical data.

ATIII, antithrombin III; PHLF, post-hepatectomy liver failure; POD, postoperative day.

**Supplementary Table 3** Spearman correlation matrix of multivariable model predictors for PHLF

| Variable | ALBI score | ICG-R15 (%) | ATIII change POD 3 |
| --- | --- | --- | --- |
| ALBI score | 1.000 | +0.322* | +0.026 |
| ICG-R15 (%) | +0.322* | 1.000 | +0.095 |
| ATIII change POD3 | +0.026 | +0.095 | 1.000 |

Values are Spearman r. *P<0.001; all other correlations: P>0.05

ALBI, albumin–bilirubin; ICG-R15, indocyanine green retention rate at 15 minutes; ATIII, antithrombin III.

**
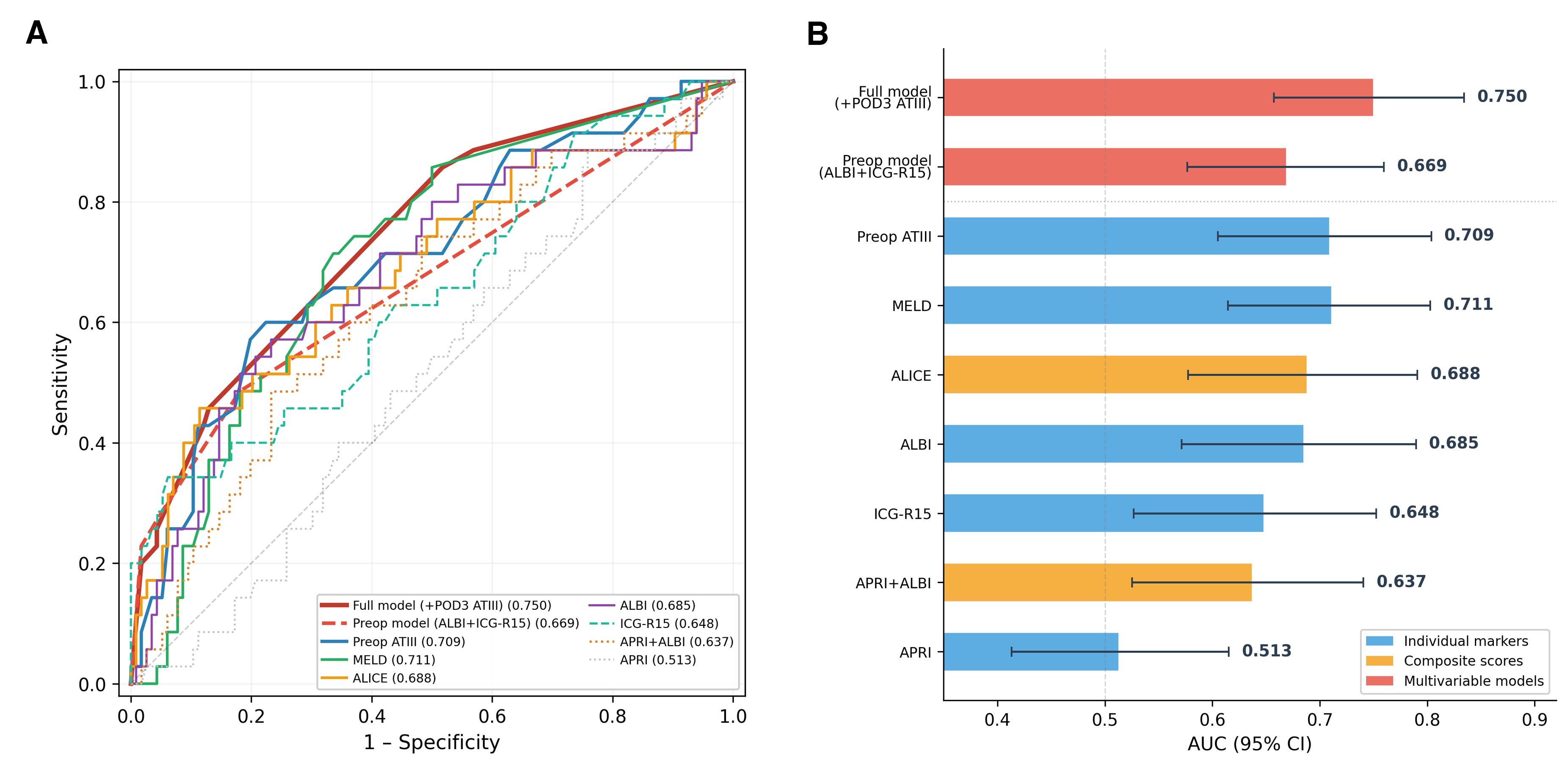
**

**Supplementary Figure 1.** Comparison of preoperative markers and scoring systems for PHLF prediction. (A) Receiver operating characteristic curves for individual preoperative markers (ATIII, ALBI, ICG-R15, MELD, APRI), composite scores (ALICE, APRI+ALBI), the preoperative-only model (ALBI grade + ICG-R15), and the full model incorporating POD 3 ATIII change ≥30%. (B) Area under the curve with 95% confidence intervals for each marker and model.

PHLF, posthepatectomy liver failure; ATIII, antithrombin III; ALBI, albumin–bilirubin; ICG-R15, indocyanine green retention rate at 15 minutes; MELD, Model for End-Stage Liver Disease; APRI, aspartate aminotransferase-to-platelet ratio index; ALICE, ALBI–ICG-R15 evaluation; POD, postoperative day; AUC, area under the curve; CI, confidence interval.

**Supplementary Table 4** Incremental predictive value of ATIII change at POD 3

| Metric | Value | 95% CI | P-value |
| --- | --- | --- | --- |
| Baseline model AUC (ALBI + ICG-R15) | 0.669 | 0.581–0.757 | — |
| Full model AUC (+ ATIII ≥30% at POD 3) | 0.750 | 0.666–0.834 | — |
| Bootstrap-corrected AUC | 0.730 | — | — |
| ΔAUC (DeLong) | +0.075 | 0.022–0.143 | 0.016 |
| NRI (continuous, rank-based) | +0.583 | 0.257–0.895 | <0.001 |

—, not applicable. 95% CI for AUC estimated by the DeLong method. P-value for ΔAUC derived from the DeLong test; P-value for NRI derived from the permutation test. Bootstrap-corrected AUC represents the optimism-corrected estimate from 1,000 bootstrap resamples. ΔAUC was estimated using the DeLong method based on paired continuous model predictions and may therefore differ from the arithmetic difference between individually rounded AUC values.

ATIII, antithrombin III; POD, postoperative day; CI, confidence interval; AUC, area under the curve; ΔAUC, change in AUC; ALBI, albumin–bilirubin; ICG-R15, indocyanine green retention rate at 15 minutes; NRI, net reclassification improvement.

**
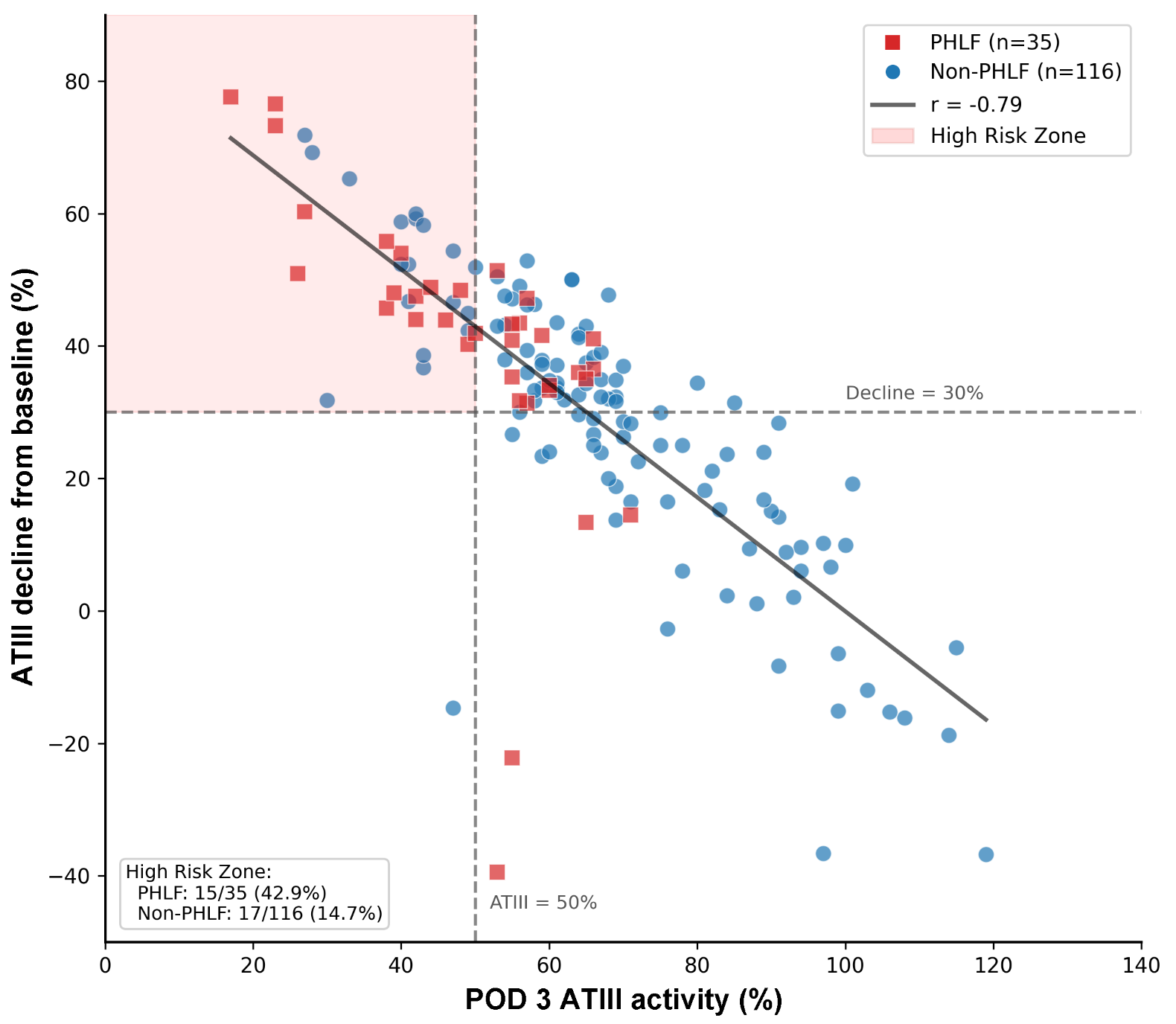
**

**Supplementary Fig. 2** Scatter plot showing the relationship between POD 3 ATIII activity and decline from baseline. Scatter plot showing POD 3 ATIII activity (%) versus percentage decline from baseline (%) in the PHLF (red squares, n=35) and non-PHLF (blue circles, n=116) groups. A strong negative correlation was observed (r = –0.79, P<0.001). The "high-risk zone" (shaded area) was defined as ATIII <50% with decline ≥30%; 42.9% (15/35) of PHLF patients fell within this zone compared to 14.7% (17/116) of non-PHLF patients.

ATIII, antithrombin III; PHLF, post-hepatectomy liver failure; POD, postoperative day.
